# Predicting in silico electron ionization mass spectra using quantum chemistry

**DOI:** 10.1186/s13321-020-00470-3

**Published:** 2020-10-20

**Authors:** Shunyang Wang, Tobias Kind, Dean J. Tantillo, Oliver Fiehn

**Affiliations:** 1 West Coast Metabolomics Center, UC Davis Genome Center, University of California, 451 Health Sciences Drive, Davis, CA 95616 USA; 2 Department of Chemistry, University of California, 1 Shields Ave, Davis, CA 95616 USA

**Keywords:** Quantum chemistry, Similarity score, Mass spectra, QCEIMS

## Abstract

Compound identification by mass spectrometry needs reference mass spectra. While there are over 102 million compounds in PubChem, less than 300,000 curated electron ionization (EI) mass spectra are available from NIST or MoNA mass spectral databases. Here, we test quantum chemistry methods (QCEIMS) to generate in silico EI mass spectra (MS) by combining molecular dynamics (MD) with statistical methods. To test the accuracy of predictions, in silico mass spectra of 451 small molecules were generated and compared to experimental spectra from the NIST 17 mass spectral library. The compounds covered 43 chemical classes, ranging up to 358 Da. Organic oxygen compounds had a lower matching accuracy, while computation time exponentially increased with molecular size. The parameter space was probed to increase prediction accuracy including initial temperatures, the number of MD trajectories and impact excess energy (IEE). Conformational flexibility was not correlated to the accuracy of predictions. Overall, QCEIMS can predict 70 eV electron ionization spectra of chemicals from first principles. Improved methods to calculate potential energy surfaces (PES) are still needed before QCEIMS mass spectra of novel molecules can be generated at large scale.

## Introduction

Mass spectrometry is the most important analytical technique to detect and analyze small molecules. Gas chromatography coupled to mass spectrometry (GC/MS) is frequently used for such molecules and has been standardized with electron ionization (EI) at 70 eV more than 50 years ago [1]. Yet, current mass spectral libraries are still insufficient in breadth and scope to identify all chemicals detected: there are only 306,622 EI-MS compound spectra in the NIST 17 mass spectral database [2], while PubChem has recorded 102 million known chemical compounds of which 14 million are commercially available. That means there is a large discrepancy between compounds and associated reference mass spectra [3]. For example, less than 30% of all detected peaks can be identified in GC–MS based metabolomics [4]. To solve this problem, the size and complexity of MS libraries must be increased. Several approaches have been developed to compute 70 eV mass spectra, including machine learning [5, 6], reaction rule-based methods [7] and a method based on physical principles, the recently developed quantum chemical software Quantum Chemical Electron Ionization Mass Spectrometry (QCEIMS) [8].

**Fig. 1:**
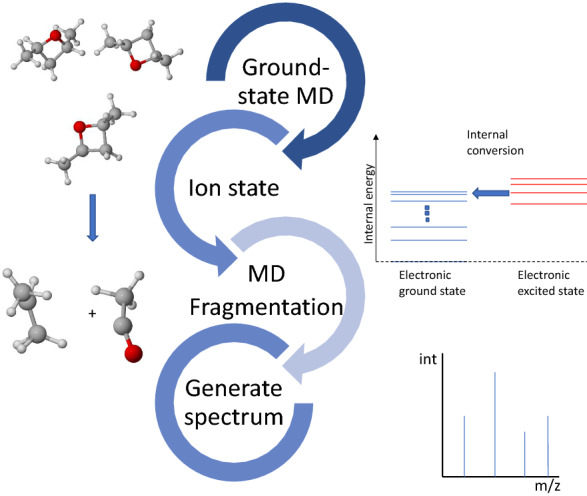
Workflow of QCEIMS. (1) generating conformers by equilibrium molecular dynamics; (2) ionizing each neutral starting structure by assigning impact excess energy (IEE) to kinetic energy; (3) generating EI fragments by parallel molecular dynamics; (4) assigning charges on each fragment using ionization potential (IP) energies and peak intensity counts, then assembling fragments to obtain summary spectra

While empirical and machine learning methods depend on experimental mass spectral data for development, quantum chemical methods only consider physical laws. Thus, in principle, QCEIMS can compute spectra for any given compound structure. Yet, approximations and parameter estimations are needed to allow predictions in a timely manner, reducing the accuracy of QCEIMS predictions. QCEIMS uses Born–Oppenheimer molecular dynamics (MD) to calculate fragment ions within picosecond reaction times with femtosecond intervals for the MD trajectories. A statistical sampling process is used to count the number of observed fragments and to derive the peak abundances for each observed ion [9] (Fig. 1).

It is unclear how reliable QCEIMS predictions are because the methods have not yet been tested on hundreds of compounds. MS matching accuracy is neither easily predictable nor quantifiable, because theoretical and experimental EI mass spectra have not been compared on a large scale. To test how structural constraints affect prediction accuracies, we utilized the QCEIMS method to predict spectra of 451 compounds with different molecular flexibility, sizes and chemical classes.

## Methods

### Molecular structure preparation

We used ChemAxon’s [10] MarvinView and MarvinSketch (v18.23) to manipulate structures. First, small molecules were manually chosen from the NIST 17 mass spectral database. 3-D coordinates were generated using the Merck Molecular Force Field (MMFF94) [11] with Avogadro (v1.2.0) [12] in Molfiles (*.mol) format. We used OpenBabel (v2.3.90) [13] to convert structures to the TurboMole format (*.tmol) as required by the QCEIMS (v2.16) program. We used the QCEIMS plotms program to export JCAMP-DX mass spectra. External additional conformers were generated independently by conformational search packages, including GMMX from Gaussian [14], the conformer generator in ChemAxon’s MarvinSketch and by using RDKit [15] (v2019.03.1).

### Parallel cluster calculation with QCEIMS

We utilized the QCEIMS program for in silico fragmentation with the following parameters: 70 eV ionization energy, 500 K initial temperature and 0.5 femtosecond (fs) time steps. For molecular dynamics, we used the semiempirical OM2 method [16] (Quantum-Chemical-Orthogonalization-Corrected Method) using the MNDO99 (v2013) [17] software. The impact excess energy (IEE) satisfied the Poisson type distribution. The Orca software (3.0.0) [18] was employed to calculate the vertical SCF ionization potential at the PBE0 [19] – D3 [20] /SV(p) [21] level.

We conducted QCEIMS calculations on cluster nodes equipped with two Intel Xeon E5-2699Av4 CPUs, 44 cores and 88 threads in total, operated at 2.40 GHz. Each node was equipped with 128 GByte RAM and a 240 GByte Intel DCS3500 datacenter grade SSD. In order to conduct and monitor the calculation process, we developed a SLURM job script to submit batch jobs. While the initial ground state molecular dynamics simulation is only single-threaded, all subsequent calculations were massively parallelized. Because QCEIMS executes multiple trajectory calculations at once, we oversubscribed the parallel number of CPU threads to be used to 66 (instead of 44) during QCEIMS production runs. Such a CPU oversubscription is possible, because molecular dynamics (OM2 with MNDO99) and density functional theory (DFT) calculations are executed in a heterogeneous way by different programs [8]. The speed advantage of using more threads than CPU cores available was confirmed with benchmarks.

### Similarity score evaluation

QCEIMS generated several outputs and logging files, including the in silico mass spectrum in JCAMP exchange format (*.jdx), structures of fragments (*.xyz) and molecular dynamics trajectories (*.xyz). We then used experimental mass spectra from the NIST17 database as references to compare with our computational results. In GC–MS, mass spectral similarity scores (0 to 1000) describe how well experimental spectra match recorded library spectra [22, 23]. Here we used the same principle for QCEIMS-generated spectra as input. We used two different kinds of similarity scores (see Eqs. 1–3):

1 $$Cos = \sqrt {\frac{{(\sum {I_{U} I_{L} } )^{2} }}{{\sum {I_{{_{L} }}^{2} \sum I_{U}^{2} } }}}$$

2 $$Dot = \sqrt {\frac{{(\sum {W_{U} W_{L} } )^{2} }}{{\sum {W_{L}^{2} } \sum {W_{U}^{2} }^{ } }}}$$

3 $$W = \left[ {Peak \;intensity]^{m} } \right[Mass]^{n}$$

We wrote a Python (v3.6) script to read mass spectra and analyze the similarity by (a) cosine similarity (*Cos*, Eq. 1) (b) weighted dot-product similarity (*Dot*, Eq. 2); with the test data, we set the parameters as m = 0.6 and n = 3. Our method calculates very similar values as implemented in the NIST MS Search program (see Additional file 1). To validate some of our simulations, we also used MassFrontier 7.0 [7] to generate fragmentation pathways and compared them with the mechanisms found from our trajectories. MassFrontier can predict fragmentation pathways from general fragmentation rules and mechanisms recorded in its literature database.

### Flexibility analysis

To describe molecular flexibility, we used two molecular descriptors: the number of rotatable bonds (RBN) [25] and Kier flexibility index (PHI) [26]. The RBN is the number of bonds for which rotation around themselves is expected to be associated with low (< 5 kcal/mol) barriers, excluding ring bonds and amide bonds. The Kier flexibility index is a structure-based property calculated from atom numbers, rings, branches and covalent radii. With fewer rotatable bonds and lower Kier flexibility index, the molecule has less conformational flexibility. The software AlvaDesc [27] (v1.0.8) is utilized to compute these properties. We used both Microsoft EXCEL for Mac and Matplotlib (v3.1.1) to analyze and visualize the data.

## Results

### Comparison of in silico and experimental spectra of example molecules

**Fig. 2:**
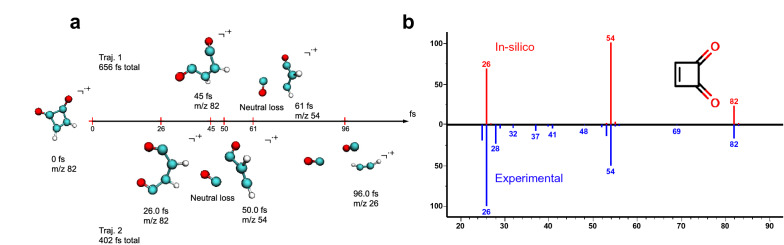
Example for correctly predicting experimental EI mass spectra through molecular dynamics. **a** Fragmentation trajectories of 3-cyclobutene-1,2-dione to generating EI fragment m/z 54 (upper panel) and m/z 26 (lower panel). **b** Quantum chemistry molecular dynamics in silico spectrum (upper panel) versus experimental mass spectrum (lower panel)

Following the general workflow, we first tested the QCEIMS software on two trajectories for a simple molecule, 3-cyclobutene-1,2-dione (Fig. 2). The observed fragment ions yielded an excellent weighted dot-product similarity score of 972 and a cosine similarity of 839. When analyzing the trajectories to show the fragmentation pathways, we found clear evidence of the mechanisms by which the three main product ions observed in the experimental mass spectrum were produced (m/z 82, 54, 26), i.e., molecular ion, a neutral loss of carbon monoxide [M-CO]˙^+^ and loss of another carbon monoxide to yield [M-2CO]˙^+^ (Fig. 2). Trajectory 2 lasted only 402 fs until the maximum of three fragments per trajectory was achieved (set in the QCEIMS source code), while trajectory 1 lasted 656 fs, because the initial two fragments reached a stable state and did not fragment further for a long time. The QCEIMS predictions also agreed with mechanisms predicted by the heuristic rule-based commercial MassFrontier software, showing first an α-cleavage followed by a CO molecule loss. This simple example shows that QCEIMS can generate correct molecular fragments and predict reasonable reaction mechanisms.

**Fig. 3:**
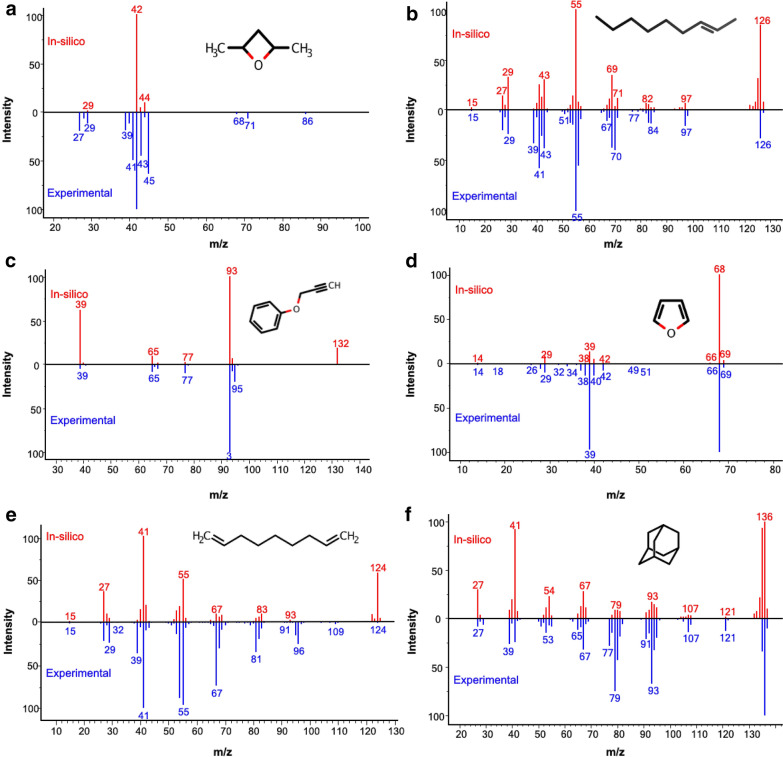
Examples for comparing experimental 70 eV EI mass spectra (lower panels) to QCEIMS in silico mass spectra (upper panels) for six small molecules

Here we show six molecules (Fig. 3a–f) as examples for QCEIMS predicted spectra versus experimental library spectra (Table 1). These examples demonstrate that QCEIMS yields different prediction accuracies. The examples also show different degrees of molecular flexibility. For each molecule, spectra showed specific characteristics that are here explained in brief.

**Table 1:** Mass spectral similarities of QCEIMS simulations against experimental spectra for select compounds

Name	InChIKey (short)^a^	M.W.^b^	RBN	PHI	Dot	Cos
2,4-Dimethyl-oxetane	KPPWZEMUMPFHEX	86.07	2	2.64	414	729
2-Nonene	IICQZTQZQSBHBY	126.27	5	7.52	789	762
2-Propynyloxy benzene	AIQRJSXKXVZCJO	132.06	0	1.17	379	426
Furan	YLQBMQCUIZJEEH	68.08	0	0.55	988	806
1,8-Nonadiene	VJHGSLHHMIELQD	124.25	6	7.05	163	713
Adamantane	ORILYTVJVMAKLC	136.13	0	1.18	883	678

^a^First 14-characters of full InChIKey

^b^M.W. is the molecular weight in Daltons (Da); RBN (rotatable bond number) and PHI (Kier flexibility index) are rigidity descriptors and Dot and Cos are mass spectral similarity scores

#### 2,4-dimethyl-oxetane (Fig. 3a)

With a weighted dot-product score of 417, this spectrum represents a low quality in silico prediction. We need to clarify that, for simplicity, we only calculated the spectrum of cis-2,4-dimethyl-oxetane, while its reference spectrum in NIST 17 mass spectral library contains no stereochemistry information because neither EI-MS nor chromatography technology can easily differentiate diastereomers. The experimental spectrum showed a low-intensity [M]˙^+^ at m/z 86 and initial neutral losses of a methane and water molecule (m/z 71 and m/z 68). QCEIMS did not predict these initial losses. Indeed, the high number of experimental fragment ions suggest that this molecule splits readily along multiple reaction pathways, most likely through breaking the molecular ether-bonds that subsequently break into smaller fragments. The main fragment ions at *m/z* 42 and m/z 44 were correctly predicted by QCEIMS as C_3_H_6_˙^+^ and C_2_H_4_O˙^+^ but not by the rule-based software MassFrontier. This case suggests that quantum mechanics-based simulations can produce novel reaction pathways that are absent from rule-base software predictions.

#### 2-Nonene (Fig. 3b)

The in silico spectrum of 2-nonene was highly similar to the experimental spectrum with dot-product match of 789. The main fragment ion at m/z 55 and the [M]˙^+^ at m/z 126 were very well reproduced. However, ion abundances of [M-1]^+^, [M-2]˙^+^, [M-3] ^+^ and [M-4]˙^+^ were overestimated. In QCEIMS, these ions resulted from loss of several atomic or molecular hydrogens, suggesting that these bonds were fragmented more easily under semiempirical methods [23] than under experimental conditions.

#### Aromatic systems (Fig. 3c and d)

Both 2-propynyloxy benzene and furan were aromatic oxygen-containing molecules with low PHI values (1.71 and 0.55, respectively). Although the presence of most fragment ions was correctly predicted by QCEIMS for both molecules, dot-product similarity scores were radically different with a dot-product of 379 for 2-propynyloxy benzene and a dot-product similarity of 988 for furan). For 2-propynyloxy benzene, this low matching score was caused by the absence of an experimental [M]˙^+^ at m/z 132 that was largely overestimated in the in silico spectrum. The fragmentation base ion (at 100% intensity) at m/z 93 represents the stable phenol ion and a neutral loss of C_3_H_3_˙ while the experimentally observed fragment at m/z 95 was missed in the QCEIMS prediction. At the same time, the presence of the C_3_H_3_^+^ product ion at m/z 39 (and a neutral loss of a phenolic radical) was overestimated by the QCEIMS method. This result suggests that the QCEIMS method needs further optimization in predicting the correct assignment of cation stability and assignment of the molecule with the lowest ionization energy in the fragmentation process (Stevenson’s rule [28]).

#### 1,8-nonadiene (Fig. 3e)

For this molecule, a great disagreement between the cosine similarity score of 713 and the weighted dot-product of 163 was observed. The weighted dot-product emphasizes high m/z ions that are penalized if missing in spectral matching. Again, QCEIMS overestimated the abundance of the molecular ion [M]˙^+^ and of several atomic or molecular hydrogens from it. In addition, QCEIMS underestimated a neutral methyl radical loss (to m/z 109) and a neutral loss of ethylene (to m/z 96). To capture all potential fragmentations in QCEIMS such as the missed ethylene loss, more accurate PES estimates are needed.

#### Adamantane (Fig. 3f)

Adamantane is a well-known inflexible molecule. Our QCIEMS simulations correctly predicted the structure of the m/z 79 product ion as protonated benzene, proved by an independent publication of an infrared multiphoton dissociation spectrum [29] and DFT computations [30]. In comparison, the rule-based MassFrontier generated less reasonable fragment molecules that included cyclopropyl-moieties. The QCEIMS results showed that the m/z 93 product ion is likely associated with both *ortho*- and *para*-protonated toluene, in accordance with infrared multiphoton dissociation spectrum results [29]. These instances highlight the ability of QCEIMS to predict non-obvious mechanisms, such as rearrangements from sp^3^ hybrid carbons to aromatic system.

### Probing the QCEIMS parameter space

A number of parameters can be chosen in the QCEIMS software, including the number of trajectories, impact excess energy per atom and initial temperatures. Other parameters such as the type of energy distribution and maximum MD time were excluded because they were already optimized during the development of QCEIMS [8]. We used OM2 because other semiempirical methods had been shown previously to perform worse [8]. For each molecule we chose one conformer and performed QCEIMS simulations with different parameter settings. By repeating QCEIMS simulations 50 times, we confirmed that identical mass spectra were obtained when using the same conformer under the same parameter settings. We changed parameter settings for 2,4-dimethyl-oxetane, 2-nonene and adamantane.

1. Number of trajectories (ntraj)

**Fig. 4:**
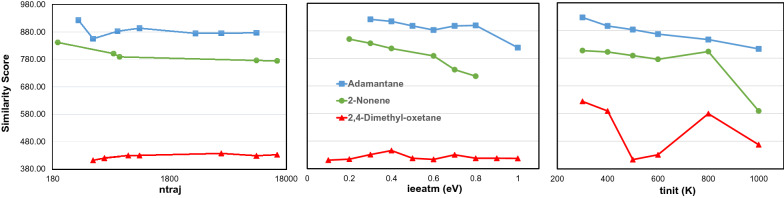
Impact of QCEIMS parameter settings on MS similarity scores comparing in silico spectra to experimental spectra. Left panel: altering the number of trajectories (ntraj). Mid panel: altering the external energy per atom (ieeatm). Right panel: altering the initial temperature (tinit)

In molecular dynamics, different reaction trajectories must be explored to cover possible routes of independent fragmentations across the energy surface. Each trajectory requires computational time, and therefore, the number of trajectories should be as low as possible. However, it is not clear a priori how many trajectories sufficiently cover the chemical reaction space and allow convergence to a consensus spectrum. By default, the QCEIMS program automatically calculates the number of trajectories by multiplying the number of atoms by 25. We explored this default value ranging from 8 to 1000 trajectories per atom for the different molecules, yielding up to 15,000 trajectories in total (Fig. 4a). For each of the three molecules, the difference between the best and the worst similarity score differed only by 10% or less. None of the three molecules had improved similarity scores with higher number of trajectories. Indeed, it appeared that increasing the number of trajectories might lead to slightly lower dot-product similarity scores as observed for 2-nonene and adamantane, possibly due to a higher contribution of rare fragmentation reactions that lead to low abundant fragment ions that negatively impact similarity to experimental spectra. We concluded that the default value of 25 trajectories per atom number in a molecule was reasonable.

(2) Impact excess energy per atom (ieeatm)

Next we tested the impact excess energy (IEE) that is introduced by the colliding electron in electron ionization as vibrational energy into the molecules. The default value (ieeatm) in QCEIMS software is set at 0.6 eV per atom on the basis of previous OM2 tests [31]. At the beginning of each molecular dynamics simulation the molecule is heated by increasing the atom velocities until the impact excess energy is converted to kinetic energy that leads to bond fragmentation. In other words, the collision energy is used to vibrationally excite and break the molecule. Higher impact excess energy will lead to a higher kinetic energy, causing the molecule to fragment more easily and to decrease the intensity of molecular ions. We observed that QCEIMS-simulated mass spectra contained fewer fragment ions than their experimental references. For example, the experimental spectrum of 2,4-dimethyl-oxetane (Fig. 3a) has 23 product ions, while our QCEIMS simulation produced only four fragment ions plus the molecular ion peak m/z 86. We probed different internal excess energies from 0.2 to 0.8 eV (Fig. 4b). With increasing IEE, more fragmentation occurs, increasing the intensity of low mass fragments, but we did not see an increase in the total number of fragments produced. Because the weighted dot-product score gives more weight to the more selective masses found at high m/z ranges, we found that higher IEE values led to decreasing similarity scores. In short, changing ieeatm did not provide a route to improve QCEIMS spectra and we kept the default value of 0.6 eV for subsequent tests.

(3) Initial temperature (tinit)

Last, we investigated the effect of temperature settings ranging from initial temperatures (tinit) of 300 to 1000 K, while keeping all other parameters at default values (Fig. 4c). For 2-nonene and adamantane we found that the initial temperatures led to decreasing similarity scores, consistent with the concept that molecules under higher temperature will have more kinetic energy and tend to fragment more easily. For QCEIMS simulations, 2,4-dimethyl-oxetane generated the molecular ion m/z 86 only at low tinit of 300 K, leading to an artificially higher similarity score. As the other two tested molecules also showed their best spectrum similarities at tinit 300 K, we chose this parameter value for a final test that utilized a combination of each best setting of ieeatm, ntraj and tinit for each molecule. Interestingly, these simulations did not lead to significant improvements or even to overall decreased similarity scores (see Additional file 1). Therefore, we kept the overall default parameter values for subsequent studies.

### Different starting conformers as input for QCEIMS

**Fig. 5:**
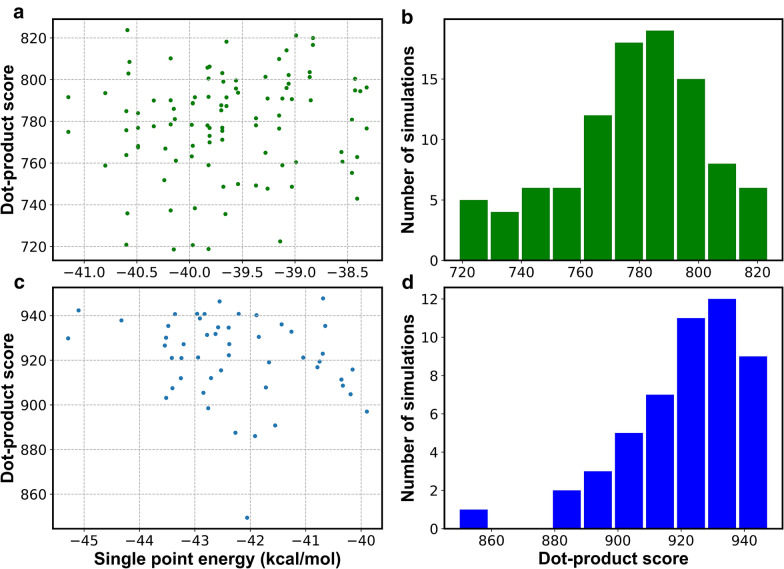
Impact of using different starting conformational isomers on MS similarity scores comparing in silico spectra to experimental spectra. Each conformer has a specific single-point electronic energy. Upper panels: 2-nonene conformers yielding dot-product MS similarity scores with histogram of the simulation results. Lower panels: adamantane conformers yielding dot-product MS similarity scores with histogram of the simulation results

Local minima on the potential energy surface that are related by rotations around single bonds are called conformational isomers, or conformers. In a mass spectrometer, the conformations of a large cohort of individual chemical molecules are distributed in accord with a Boltzmann distribution at a given ion source temperature. All conformers contribute to the final mass spectrum, to varying degrees related to their relative energies. Ideally, QCEIMS should cover the overall ensemble of conformers. To investigate the impact of the input conformers on the overall QCEIMS results, we selected the highly flexible 2-nonene (PHI = 7.51, RBN = 5) and the non-flexible adamantane (PHI = 1.17, RBN = 0) structures. We employed the GMMX software with the Merck Molecular Force Field (MMFF94) to generate starting conformers for individual QCEIMS simulations. For 99 simulations with different starting conformers of 2-nonene, the maximum difference between the lowest-energy and the highest-energy conformer was 2.83 kcal/mol (Fig. 5a). For these conformers, dot-product similarity scores ranged from 719 to 824, with a median of 781 and a standard deviation of 24 (Fig. 5b). Due to the rigid skeleton and inflexibility of adamantane, GMMX provided only one conformer. Therefore, we used the open source molecular dynamics package CP2K [32] to generate 50 adamantane structures with twisted or stretched bonds that yielded an overall energy range of 5.39 kcal/mol (Fig. 5c). Dot-product similarity scores ranged from 849 to 948, with a median similarity of 923 and a standard deviation of 31 (Fig. 5d). The examples of these very different molecules showed that QCEIMS similarity scores were independent from input conformer energies (Fig. 5a, c). Yet, these examples also showed that for both molecules, the QCEIMS fragmentation of specific conformers can lead to quite different dot-product similarities compared to experimental mass spectra, ranging over 100 similarity score units. In addition, we found that dot-product similarities were not normally distributed (Fig. 5b, d). Our results showed that conformational and other small structural changes may affect QCEIMS simulations. Although adamantane has only a single conformational energy minimum, even slight bond stretches or twists led to quite different mass spectral similarity scores, presumably by biasing molecular dynamics trajectories toward different regions of the potential energy surface. While the QCEIMS software automatically chooses energy-optimized conformers, we propose that a range of different conformers must be calculated to get a good estimate of average mass spectra across the conformational space.

### Large scale QCEIMS prediction of small molecule fragmentations

In order to be useful for experimental mass spectrometry, in silico predictions must not only correctly explain fragmentation and rearrangement reactions for specific molecules, but must also be scalable to generate spectra for hundreds, if not thousands of molecules. Here, we demonstrate the scalability of QCEIMS predictions for small molecules to systematically evaluate parameters and overall accuracies.

**Fig. 6:**
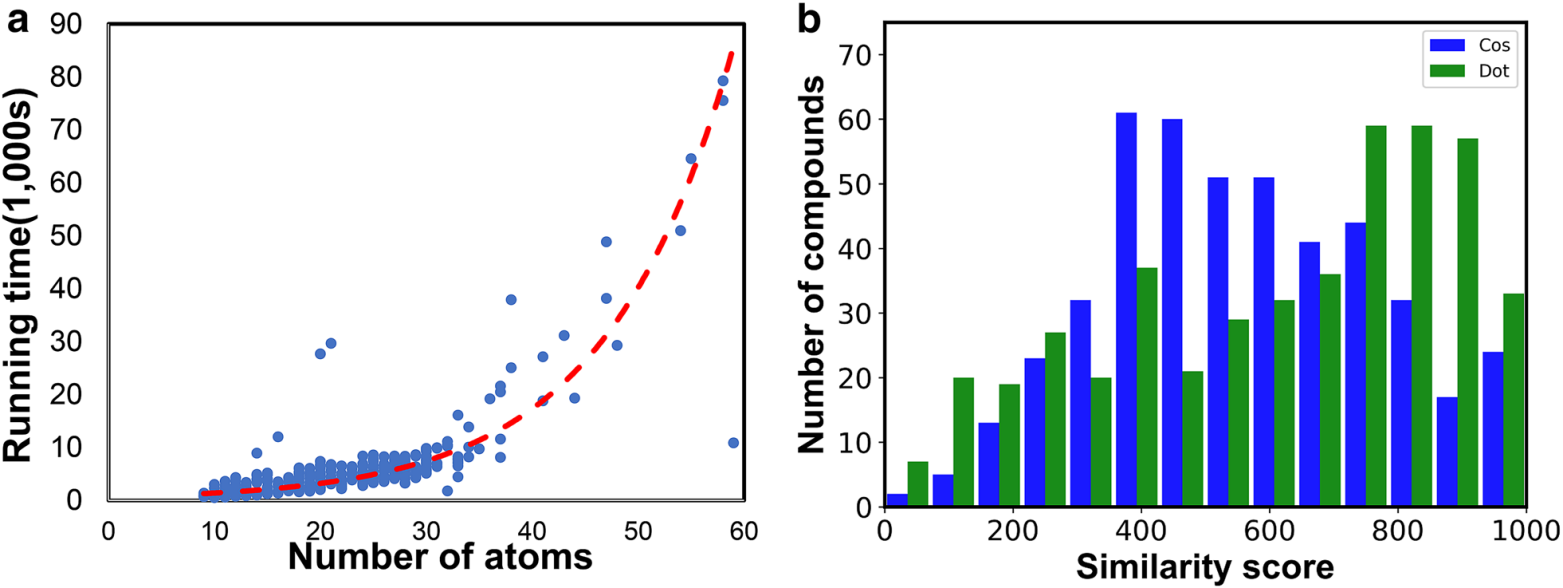
**a** Processing time of QCEIMS simulations of all 451 molecules versus the number of atoms per molecule. Red trend line: fitted exponential functions. **b** Histogram of weighted dot-product MS similarity scores against experimental spectra for all 451 molecules versus simple cosine similarity matches

The OM2 method only supports carbon, hydrogen, nitrogen, oxygen and fluorine. We therefore chose 451 low molecular weight compounds containing only carbon, hydrogen, nitrogen and oxygen (CHNO). Molecular masses ranged from 26 to 368 Da with an average mass of 129 Da (see Additional file 1). For OM2, computational effort scales as O(N^2^) ~ O(N^3^) [33], with N as number of atoms per molecule [33]. The number of single point energy calculations can be estimated to be linearly related to the number of trajectories, and thus linear to the number of atoms. On our computer system with 66 CPU threads, we achieved an average calculation time of 1.55 h per molecule (Fig. 6a). Yet, as expected, calculation times exponentially increased with the number of atoms per molecule. For example, with more than 50 atoms, calculation times exceeded 14 h on the system we had employed (Fig. 6a).

Overall, the QCEIMS calculations across all 451 molecules yielded moderately accurate weighted-dot product similarity scores with an average of 608 (Fig. 6b) [24]. In GC–MS, similarity scores below 500 are usually not considered for annotation of compounds. While similarity scores above 700 may represent true matches, only scores above 850 are regularly used for direct compound identifications in GC–MS experiments [24]. 47% of all molecules showed good dot-product match factors > 700 and 20% of the molecules had excellent scores at > 850 similarity. In comparison, lower cosine similarity scores were achieved with an average mass spectral similarity of 557 and a much higher proportion of unacceptably low scoring spectra at similarities < 500 (Fig. 6b). The regular cosine similarity score does not use weight functions for specific m/z values, unlike the weighted dot product score introduced in 1994 [22] that gives more weight to more specific high m/z product ions in MS fragmentation compared to less specific low m/z fragmentations based on large GC–MS library evaluations. Here, we see a similar trend for QCEIMS spectra.

### Molecular descriptors and prediction accuracy

Next we tested the impact of the chemical structures themselves. We used ClassyFire [34] to classify all 451 chemicals into superclasses (supplement file). We found QCEIMS predictions were significantly worse when comparing the organic oxygen superclass of 75 compounds against other superclasses with more than 50 members. Organic oxygen compounds had an average weighted dot-product of 520 whereas the 128 organoheterocyclic compounds achieved significantly better similarities of 648 at p < 0.0015 (supplement file). The 100 organic nitrogen compounds yielded an average dot-product similarity of 657 at p < 0.001 and the 62 hydrocarbons gave an average of dot-product similarity of 692 at p < 0.0001 (supplement file). In conclusion, the QCEIMS method appears to perform worse for oxygen-containing organic compounds than for other major classes. For superclasses with fewer than 50 compounds, statistical tests were deemed to be not robust enough to allow such conclusions.

**Fig. 7:**
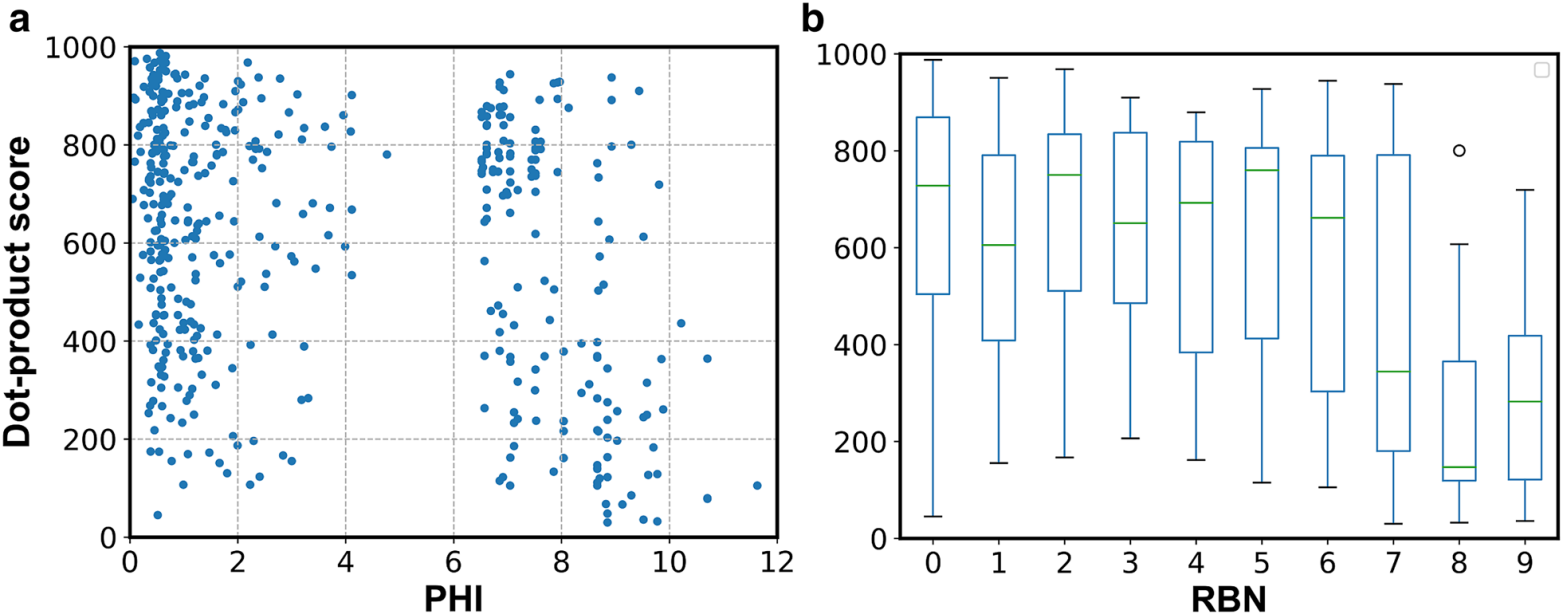
Impact of molecular flexibilities on MS similarity scores comparing in silico spectra to experimental spectra. Influence of molecular flexibility. **a** Scatter point plot of dot-product scores versus Kier flexibility index PHI; **b** boxplot of dot-product scores versus rotational bond number RBN

We also tested if rigid molecules resulted in better prediction accuracy than more flexible ones. Our hypothesis was based on an initial observation that for planar aromatic compounds such as pyridine or aniline, QCEIMS created better quality spectra than for molecules with long chain flexible structures. Our compound data set contained 295 molecules with low flexibility at Kier flexibility index (PHI) < 5 and 161 molecules with high flexibility of PHI > 6. Dot product scores varied significantly across both high-flexibility and low-flexibility molecules (Fig. 7a). We found no relationship between flexibility and prediction accuracy. Similarly, we tested rotatable bond number (RBN) as a potential cause for prediction errors (Fig. 7b). The median scores for molecules with different RBN values varied between 200 and 800 and did not depend on increasing RBN. This finding suggests that prediction accuracy is independent of the number of rotatable bonds. In conclusion, we could not find a correlation between flexibility and prediction accuracy at the level of simulation employed.

## Conclusions

We here show that quantum chemistry calculations can be effectively used to correctly predict electron ionization fragmentation mass spectra as used in GC/MS analyses worldwide. Using QCEIMS software, mechanisms of fragmentation confirmed classic fragmentation rules. However, we found large differences in accuracy of predictions for different molecules. Changing parameters in QCEIMS was not a viable method to improve simulation results. Likely, capturing the potential energy surface accurately or even conducting the excited-state molecular dynamics [35, 36] can be the key to further improving EI-MS prediction. For the first time, QCEIMS simulation was tested on hundreds of small organic molecules with limited computational resources within 1 month. We found that the superclass of organooxygen compounds performed much worse than organoheterocyclic compounds, hydrocarbons or organic nitrogen compounds. This observation may lead to future improvements in QCEIMS software as well as further inclusion of other heteroatoms in QCEIMS simulations. In comparison, QCEIMS of inorganic molecules [37] were regarded as less important for GC–MS applications.

## Supplementary information

**Additional file 1.** Metadata of the in-silico MS database and raw data plots.

## Data Availability

All data is publicly available for commercial and non-commercial use from Data Supplement I (https://zenodo.org/record/3730507); II (https://zenodo.org/record/3942723); III (https://zenodo.org/record/3841707); IV (https://zenodo.org/record/3839993).

## Abbreviations

EI: Electron ionization
NIST: National Institute of Standards and Technology
MoNA: MassBank of North America
MS: Mass spectra
MD: Molecular dynamics
IEE: Ionization excess energy
PES: Potential energy surface
GC: Gas chromatography
RAM: Random access memory
CPU: Central processing unit
SSD: Solid-state drive
DFT: Density functional theory
RBN: Number of rotatable bonds
PHI: Kier flexibility index
InChIKey: Hashed counterpart of International Chemical Identifier
ieeatm: Impact excess energy (IEE)/atom
ntraj: Number of trajectories
tinit: The initial temperature of the vaporized substrate
